# Comparative genome-wide methylation analysis of longissimus dorsi muscles between Japanese black (Wagyu) and Chinese Red Steppes cattle

**DOI:** 10.1371/journal.pone.0182492

**Published:** 2017-08-03

**Authors:** Xibi Fang, Zhihui Zhao, Haibin Yu, Guangpeng Li, Ping Jiang, Yuwei Yang, Runjun Yang, Xianzhong Yu

**Affiliations:** 1 College of Animal Science, Jilin University, Changchun, People’s Republic of China; 2 The Key Laboratory of National Education Ministry for Mammalian Reproductive Biology and Biotechnology, Inner Mongolia University, Hohhot, People’s Republic of China; 3 Department of Biological Sciences, Clemson University, Clemson, SC, United States of America; International Nutrition Inc, UNITED STATES

## Abstract

DNA methylation is an important epigenetic mechanism involved in expression of genes in many biological processes including muscle growth and development. Its effects on economically important traits are evinced from reported significant differences in meat quality traits between Japanese black (Wagyu) and Chinese Red Steppes cattle, thus presenting a unique model for analyzing the effects of DNA methylation on these traits. In the present study, we performed whole genome DNA methylation analysis in the two breeds by whole genome bisulfite sequencing (WGBS). Overall, 23150 differentially methylated regions (DMRs) were identified which were located in 8596 genes enriched in 9922 GO terms, of which 1046 GO terms were significantly enriched (*p*<0.05) including lipid translocation (GO: 0034204) and lipid transport (GO: 0015914). KEGG analysis showed that the DMR related genes were distributed among 276 pathways. Correlation analysis found that 331 DMRs were negatively correlated with the expression levels of differentially expressed genes (DEGs) with 21 DMRs located in promoter regions. Our results identified novel candidate DMRs and DEGs correlated with meat quality traits, which will be valuable for future genomic and epigenomic studies of muscle development and for marker assisted selection of meat quality traits.

## Introduction

Beef, which is rich in protein, iron, zinc, B vitamins and essential polyunsaturated fatty acids, is a source of high-quality nutrition for humans [1]. However, several diseases, such as heart disease, diabetes and obesity, have been linked with the consumption of fat from beef. Thus, beef breeding program should try to find a balance between eating quality and nutritional quality, which demands a better understanding of the genetic and epigenetic mechanisms controlling meat quality traits.

Advancement in DNA sequencing technology has led to rapid accumulation of data on individual sequence variations in livestock, including cattle [2, 3], which, in combination with the development of high-density bovine genotyping based genome-wide association studies [4, 5], makes it possible for selection of production traits and increased rate of genetic progress in livestock. However, quantitative traits such as meat quality traits are generally determined by many genes, thus the benefit of a particular genetic variant in marker-assisted selection is limited to the proportion of the variant contributing to the heritable phenotypic variance. More importantly, many of the variants detected using GWAS have been in regulatory regions which result in changes in gene expression rather than protein sequences, indicating that the regulatory mechanisms are possibly epigenetics in nature. It is imperative to understand how epigenetics affects phenotypes through the regulation of gene expression. Furthermore, sensory quality of meat is affected by many environmental factors (such as nutrition, stress, exposure to pollution) that likely exert their influence through epigenetic modifications [6].

Epigenetics is the study of heritable changes in gene expression that are not caused by DNA sequence changes [7]. Epigenetic mechanisms include DNA methylation, histone modifications, and non-coding RNAs. DNA methylation plays a central role in regulating many different biological processes, such as growth, development, genomic imprinting and X-chromosome inactivation in females [8]. There is a clear link between abnormal DNA methylation and human diseases [9]. DNA methylation is a critical intermediate molecular phenotype which is considered as the linkage between genotypes and other macro-level phenotypes and maybe attributed to the missing heritability [10]. Current data suggest that genetic variation at specific loci is correlated with the quantitative traits of DNA methylation [11], and genetic variants at CpG sites (meSNPs) could cause changes in their methylation status. It is suggested in human studies that meSNPs attributed a large portion of observed signal from methylation-associated loci (meQTLs) and might be crucial in explaining the results of association between genetic variants and epigenetic changes.

In livestock, genome-wide methylation studies have been performed in chicken, pig, cattle, and sheep, especially in the high-value muscle tissues [6, 12]. The objective of the present study is to perform a comparative genome-wide methylation analysis of longissimus dorsi muscles between the Japanese Black (Wagyu) and Chinese Red Steppes cattle, especially in DMRs and DEGs. Since significant differences exist in meat quality traits between the two breeds, further correlation analysis will allow us to identify DNA methylation variants correlated with meat quality traits, which will be valuable for future genomic and epigenomic studies of muscle development and for marker assisted selection of meat quality traits.

## Materials and methods

### Ethics statement

Animal care and experiments were performed according to the guideline established by the Regulation for the Administration of Affairs Concerning Experimental Animals (Ministry of Science and Technology, China, 2004) and approved by the Animal Welfare and Research Ethics Committee at Jilin University (Approval ID: 20140310).

### Animals and samples

Three longissimus muscle (LM) tissue samples of Japanese black cattle (28 months old) and three LM samples of Chinese Red Steppes cattle (28 months old) were provided by the National Research Center for Animal Transgenic Bio-technology, Inner Mongolia University (Hohhot, China) and the Branch of Animal Science, Jilin Academy of Agricultural Sciences (Gongzhuling, China), respectively. The three Japanese black cattle were randomly chosen from 20 males born from cows that were artificially inseminated with semen stocks from the same bull. The three Chinese Red Steppes cattle were similarly chosen from 20 males with a common father. The two farms housing the two groups of cattle were located at similar altitudes with similar natural weather conditions. The cattle in both groups were raised under similar normal conditions on a diet of corn and hay with access to feed and water *ad libitum*. The tissue samples were transported in dry ice and stored in the laboratory in liquid nitrogen.

### Extraction of DNA and bisulfite conversion

DNA was extracted from longissimus muscle tissues with a DNA extraction kit (Tiangen, Beijing, China) according to the manufacturer’s protocol and the concentration of the genomic DNA was determined with a NanoDrop ND-2000 UV spectrophotometer (Thermo Fisher Scientific Inc., USA). The degradation and contamination of genomic DNA was monitored with 1% agarose gel electrophoresis.

### Library preparation and quantification

Three DNA libraries for each breed were constructed and grouped by breed as WC_LC (Wagyu) and RC_LC (Chinese Red Steppes) for subsequent analysis. 5.2 μg genomic DNA spiked with 26 ng lambda DNA were fragmented to 200–300 bp by sonication with a focused-ultra sonicator (Covaris, Woburn, MA, USA), followed by end repair and adenylation. Cytosine-methylated barcodes were ligated to sonicated DNA according to the manufacturer’s protocol. The DNA bisulfite conversion was performed using the EZ DNA Methylation Gold kit (Zymo Research, California, USA) according to the manufacturer’s instruction. After DNA bisulfite conversion, single-strand DNA fragments were PCR amplified using the KAPA HiFi HotStart Uracil + ReadyMix (2X) (Kapa Biosystems, Wilmington, MA, USA). Library size was quantified by quantitative PCR and a Qubit® 2.0 Flurometer (Life Technologies, CA, USA), and the insert size was checked on an Agilent Bioanalyzer 2100 system (Agilent Technologies, Santa Clara, CA, USA).

### Whole genome bisulfite sequencing and identification of DMRs

The libraries from 6 samples were sequenced on an Illumina Hiseq2500 platform and 125 nt paired-end reads were generated by Novogene (Novogene, Beijing, China). Read sequences were produced by the Illumina pipeline with FastQ format and were pre-processed by in-house perl scripts. At first, all parts of the 3’ adapter oligonucleotide sequences were filtered out. Secondly, if the percentage of unknown bases (Ns) was greater than 10% the read was removed. Thirdly, reads with low quality (more than 50% of bases with PHRED score< = 5) were trimmed. At the same time, Q20, Q30 and GC content were calculated. All of the subsequent analyses were based on the remaining clean reads that passed the filters.

After filtering the low quality reads, the clean reads data were aligned to the Ensembl bovine reference genome (*Bos taurus* UMD_3.1.1) and the bisulfite mapping of methylation sites were performed by Bismark (version 0.12.5) [13] with score_min L, 0, -0.2 and then indexed using Bowtie2 [14]. At first, the reference genome and clean reads were transformed into bisulfite-converted version (C-to-T and G-to-A converted). Secondly, sequence read which produced a unique best alignment from the two alignment processes (original top and bottom strand) was then compared to the normal genomic sequence and the methylation status of all cytosine positions in the reads were identified. The reads that aligned to the same regions of genome were regarded as duplicated ones. The sequencing depth and coverage were estimated using deduplicated reads. The data of methylation extractor were transformed into bigWig format for visualization using IGV browser. The sodium bisulfite non-conversion rate of bovine genome was calculated as the percentage of cytosine sequenced at cytosine reference positions in the lambda genome.

To identify the differentially methylated regions (DMRs) between Japanese black cattle (Wagyu) and Chinese Red Steppes, we referenced the modeled of Hao. Y. et al [1] to estimate methylation level [15]. DMRs were identified using a sliding-window approach of swDMR software (http://122.228.158.106/swDMR/) [16] and the window is set to 1000 bp and step length at 100 bp. Fisher test is implemented to detect the DMRs.

### GO and KEGG enrichment analysis of genes related to DMRs.

Gene related to DMRs were implemented by the GOseq R package [17], in which gene length bias was corrected. GO terms with corrected *p* values of less than 0.05 were considered significantly enriched. KOBAS software [18] was used to test the statistical enrichment of DMR-related genes in KEGG pathways [19, 20]. Pathway with a corrected *p* value <0.05 was considered as significantly enriched.

### Sample preparation for RNA‑seq analysis

Total RNA was isolated from longissimus dorsi muscles using Trizol Reagent (Invitrogen, USA) according to the manufacturer's instructions. Total RNA was treated with DNase I (NEB, Beijing, China), extracted with phenol-chloroform, and precipitated with ethanol. The quality and quantity of total RNA were determined using an Agilent 2100 Bioanalyzer (Agilent technologies, Palo Alto, CA). The mRNA was purified from total RNA using poly-T oligo-attached magnetic beads. 1.5 μg of mRNA from each sample was used to construct six cDNA libraries for sequencing. The mRNA was treated with a Thermomixer (Eppendorf AG, Hamburg, Germany) to generate fragments with an average size of 200 bp (200 ± 25 bp) for the paired-end libraries. The fragmented mRNA was then used as templates for synthesizing the first-strand cDNA. The double-stranded cDNAs were purified and ligated to adaptors for Illumina paired-end sequencing. Library concentration was quantified by qPCR and a Qubit® 2.0 Flurometer (Life Technologies, CA, USA), and the insert size was checked on an Agilent Bioanalyzer 2100 system (Agilent Technologies, Palo Alto, CA). The cDNA libraries were sequenced using the Illumina HiSeq2000 platform by the Beijing Genomics Institute (BGI) and 100 nt paired-end reads were generated.

### Quantification of gene expression levels and identification of DEGs

According to our data, the EB-Seq algorithm was applied to filter differentially expressed genes for the WC_LC and RC_LC groups and the URL of the GTF file used to annotate the EB-Seq is ftp://ftp.ensembl.org/pub/release-79/gtf/bos_taurus/Bos_taurus.UMD3.1.79.gtf.gz. After significance analysis and FDR (false discovery rate) analysis[21], we selected the DEGs according to the Log2^FC^ >0.585 or <-0.585, FDR <0.05.

### Association analysis

For association analysis, a set of differentially expressed genes with differential methylation was obtained from the intersection between the set of differentially methylated genes and the set of differentially expressed genes. Negative correlations were identified by correlation analysis between the methylation level of DMRs and the expression level of the corresponding genes (r with negative value). Due to the small size of the sample, the present data were not further screened with r^2^ and *p* values. In addition, GO analysis was performed on negatively correlated genes in Biological Process, Molecular Function, Cellular Component, their sub-categories and KEGG pathway.

## Results

### Bisulfite sequencing and DNA methylation profiling

The BS conversion rates of genomic DNA ranged from 99.61% to 99.72%, and a total of 668.37 Gbp of raw sequence data was obtained from 6 samples. After filtering out low quality data, 630.39 Gbp clean sequences were mapped to *Bos taurus* (UMD_3.1.1) with Q30 of clean full-length reads ranging from 86.15 to 90.84%. High quality methylation maps of the two bovine breeds were obtained and the unique mapping rates ranged from 69.57% to 81.82%. The details of sequencing data quality were shown in Table 1. The results of WGBS analyses identified differentially methylated regions (DMRs) covering almost the entire genome with sufficient depth and high resolution. The average distribution coverage of the genome in 6 samples was shown in S1 Fig.

**Table 1:** Sequencing data by whole genome bisulfite sequencing (WGBS) for Japanese Black cattle and Chinese Red Steppes.

Samples	Raw Bases	Clean Bases	Error Rate	Q20	Q30	GC Content	BS Conversion Rate	Total reads	Mapped reads	Mapping rate(%)
**WC_LM3**	114.15G	97.76G	0.05%	91.65%	86.15%	21.31%	99.72%	391054243	272063147	69.57
**WC_LM1**	118.16G	110.44G	0.04%	92.87%	87.74%	24.69%	99.63%	441776127	352743588	79.85
**WC_LM2**	127.22G	115.88G	0.04%	92.86%	87.41%	24.56%	99.61%	463516936	364168595	78.57
**RC_LM1**	108.14G	100.9G	0.03%	95.00%	90.72%	24.79%	99.68%	403581374	327796966	81.22
**RC_LM2**	118.51G	109.13G	0.03%	93.98%	89.10%	24.63%	99.63%	436533061	341001097	78.12
**RC_LM3**	102.19G	96.28G	0.03%	94.97%	90.84%	23.97%	99.65%	385132178	315113299	81.82

WC_LM1, WC_LM2, WC_LM3: longissimus dorsi muscle samples of Japanese black cattle (Wagyu); RC_LM1, RC_LM2, RC_LM3: longissimus dorsi muscle samples of Chinese Red Steppes.

CpGs, CHHs and CHGs (H = C, T and A) were methylated at different levels. 48.27–64.61% of CpGs in the whole genome were methylated while only 0.07–0.10% and 0.8–0.13% cytosines in the CHG and CHH contexts were methylated, respectively (Table 2). The distribution details of methylated cytosines in the contexts of CpGs, CHHs and CHGs in whole genome were shown in S1 Table.

**Table 2:** Methylation levels of all CpG sites in genomes of Japanese Black cattle and Chinese Red Steppes.

Samples	mC percent(%)	mCpG percent(%)	mCHG percent(%)	mCHH percent(%)
**WC_LM3**	2.47%	48.27%	0.07%	0.10%
**WC_LM1**	2.88%	56.74%	0.07%	0.08%
**WC_LM2**	2.93%	57.60%	0.08%	0.10%
**RC_LM1**	2.88%	56.32%	0.09%	0.12%
**RC_LM2**	3.30%	64.61%	0.10%	0.13%
**RC_LM3**	2.91%	56.81%	0.09%	0.12%

WC_LM1, WC_LM2, WC_LM3: longissimus dorsi muscle samples of Japanese black cattle (Wagyu); RC_LM1, RC_LM2, RC_LM3: longissimus dorsi muscle samples of Chinese Red Steppes. MC percentage (%): percentage of genome-wide methylated cytosines. MCpG percentage (%): percentage of methylated cytosines in CG context. MCHG percentage (%): percentage of methylated cytosines in CHG context. MCHH percentage (%): percentage of methylated cytosines in CHH context.

**Fig 1:**
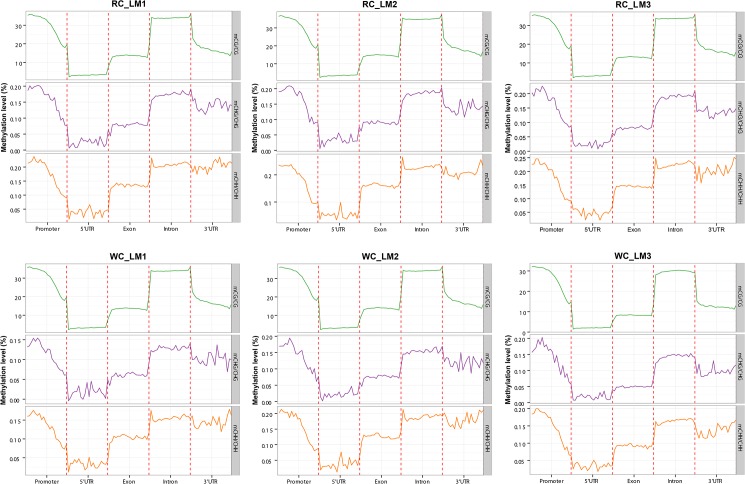
DNA methylation levels across genomic elements in Japanese black cattle (Wagyu) and Chinese Red Steppes. Abscissa represents different genomic elements, the ordinate represents the level of methylation and different colors represents different sequence contexts (CpG, CHG, CHH). WC_LM1, WC_LM2, WC_LM3: longissimus dorsi muscle samples of Japanese black cattle (Wagyu); RC_LM1, RC_LM2, RC_LM3: longissimus dorsi muscle samples of Chinese Red Steppes.

To better understand DNA methylation levels at various functional genomic elements, such as promoters, exons, introns, etc., the 2 kb region upstream of the TSS point (defined as the promoter region) of each gene was equally divided into 20 bins, and the corresponding bin level on C locus of all genes were averaged. For mC sites in each context, the average methylation levels and the genome-wide landscape of distribution of various functional genomic elements of 6 samples were built by ggplot2 R package[22] as shown in Fig 1. Similar tendencies of methylation levels in all samples were observed in different genetic elements in the three mC contexts, without any significant differences among them. Overall, the DNA methylation levels in introns were the highest, followed by exons and 3’ UTR, with 5’ UTR showing the lowest level. In promoter regions, the proximal regions had the lowest DNA methylation levels, followed by the intermediate regions, with the distal regions having the highest levels. As for mC in CHH context, the 5’ proximal regions of intron had the highest methylation levels in all samples which were different from mCs in other contexts.

**Fig 2:**
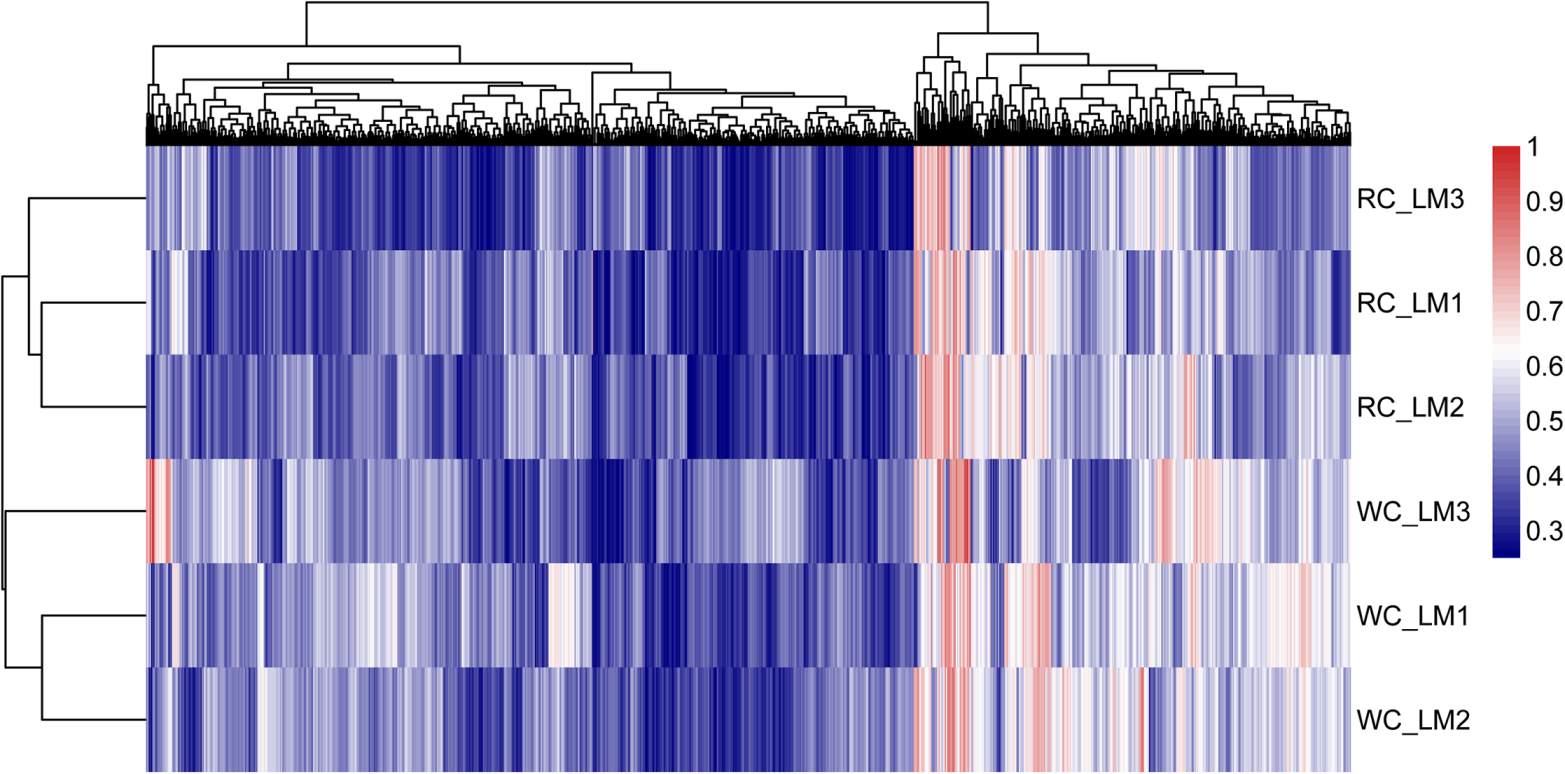
The cluster analysis of genomic methylation levels in Japanese black cattle (Wagyu) and Chinese Red Steppes. Heat map displays highly methylated loci in red and sparsely methylated loci in blue.

The difference and trend in genomic methylation levels between samples can be analyzed by regional cluster classification analysis to understand the biological significance [23]. Using hierarchical clustering method, we analyzed the whole genomic methylation in Japanese black cattle (Wagyu) and Chinese Red Steppes cattle, as shown in the heat maps of Fig 2, which revealed a clear separation between the two breeds. Our results also showed that the biological variation among the three samples within the same breed was extremely low. In addition, the cluster analysis results for the genomic methylation in different functional regions were summarized in S2 Fig.

### Characterization of DMRs

**Fig 3:**
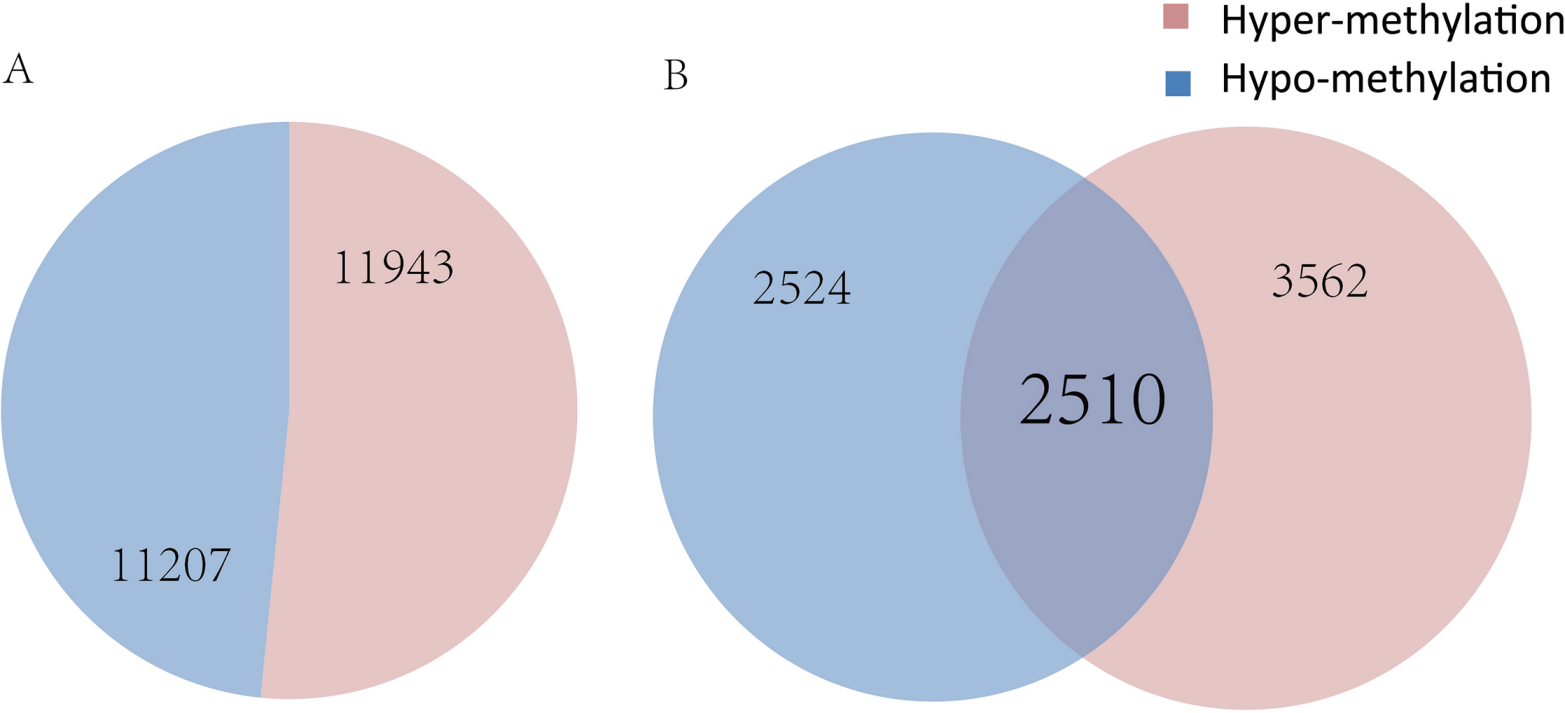
Numbers of DMRs, hyper-methylated genes, and hypo-methylated genes in Japanese black cattle (Wagyu) and Chinese Red Steppes. (A) Numbers of DMRs; (B) Numbers of genes related to DMRs. DMRs: differentially methylated regions.

In total, we compared the methylation levels of longissimus dorsi muscle from the two breeds and identified 23,150 DMRs in gene bodies or promoter regions from 8,596 genes that were significantly different between Wagyu and Chinese Red Steppes (corrected *p*< 0.05) which have different meat quality traits. Among the DMRs, 11,943 corresponded to 6,072 genes with higher methylation levels in Wagyu than that in Chinese Red Steppes, whereas 11,207 corresponded to 5,034 genes with lower methylation levels in Wagyu (Fig 3). The top 20 DMRs were listed in Table 3 by ascending order of corrected *p* value. Interestingly, 2,510 genes possessed multiple DMRs with both hyper-methylated (higher methylation level in Wagyu than in Chinese Red Steppes) regions and hypo-methylated (lower methylation level in Wagyu than in Chinese Red Steppes) regions, such as acyl-CoA synthetase long-chain family member 6 (ACSL6), epidermal growth factor receptor (EGFR), 1-acylglycerol-3-phosphate O-acyltransferase 4 (AGPAT4), upstream binding protein 1(UBP1).

**Table 3:** Top 20 DMRs based on the ratio of methylation levels between Japanese Black cattle and Chinese Red Steppes.

Chrom	Stat	Gene ID	Gene context	Gene Name
**29**	hyper	ENSBTAG00000008274	exon,intron	MUC5B
**4**	hyper	ENSBTAG00000008542	exon,intron	SSPO
**19**	hyper	ENSBTAG00000039599	exon	HOXB4
**24**	hyper	ENSBTAG00000022819	exon	ONECUT2
**3**	hyper	ENSBTAG00000000963	exon,intron,promoter	-
**5**	hyper	ENSBTAG00000013912	intron	TXNRD1
**13**	hyper	ENSBTAG00000019508	intron	SEC61A2
**3**	hyper	ENSBTAG00000015154	exon,intron	MCL1
**3**	hyper	ENSBTAG00000015154	exon,utr5,promoter	MCL1
**17**	hyper	ENSBTAG00000010464	promoter	MN1
**1**	hypo	ENSBTAG00000009942	intron	PLCL2
**18**	hypo	ENSBTAG00000009364	exon,intron	-
**18**	hypo	ENSBTAG00000009364	exon	-
**18**	hypo	ENSBTAG00000009364	exon,intron,promoter	-
**9**	hypo	ENSBTAG00000039329	intron	RAET1G
**9**	hypo	ENSBTAG00000047902	intron	-
**17**	hypo	ENSBTAG00000014111	intron	INPP4B
**3**	hypo	ENSBTAG00000038025	promoter	H2B
**3**	hypo	ENSBTAG00000038476	exon	HIST2H2AB
**3**	hypo	ENSBTAG00000040098	exon	-

DMRs: differentially methylated regions; chrom: location of DMRs on chromosomes; stat: status of methylation levels; hyper: Japanese Black cattle have higher methylation levels than Chinese Red Steppes; hypo: Japanese Black cattle have lower methylation levels than Chinese Red Steppes; promoter: the 2kb region upstream of the TSS point.

The length of DMRs ranged between 3 nt to 3,780 nt (S3 Fig). Most of the DMRs were located at introns (73.66%), followed by exons (14.36%) and regulatory regions (11.98%), such as promoters, 5’UTRs and 3’UTRs, as shown in Table 4.

**Table 4:** DMR distribution among different genetic elements in Japanese Black cattle and Chinese Red Steppes.

Gene context	Hyper-methylated	Hypo-methylated
PROMOTER	1581	793
5’ UTR	351	92
EXON	2478	1599
INTRON	10461	10444
3’ UTR	309	273

DMRs: differentially methylated regions; gene context: functional region names; promoter: the 2kb region upstream of the TSS point.

### Gene ontology and KEGG enrichment analyses of genes related to DMRs.

**Fig 4:**
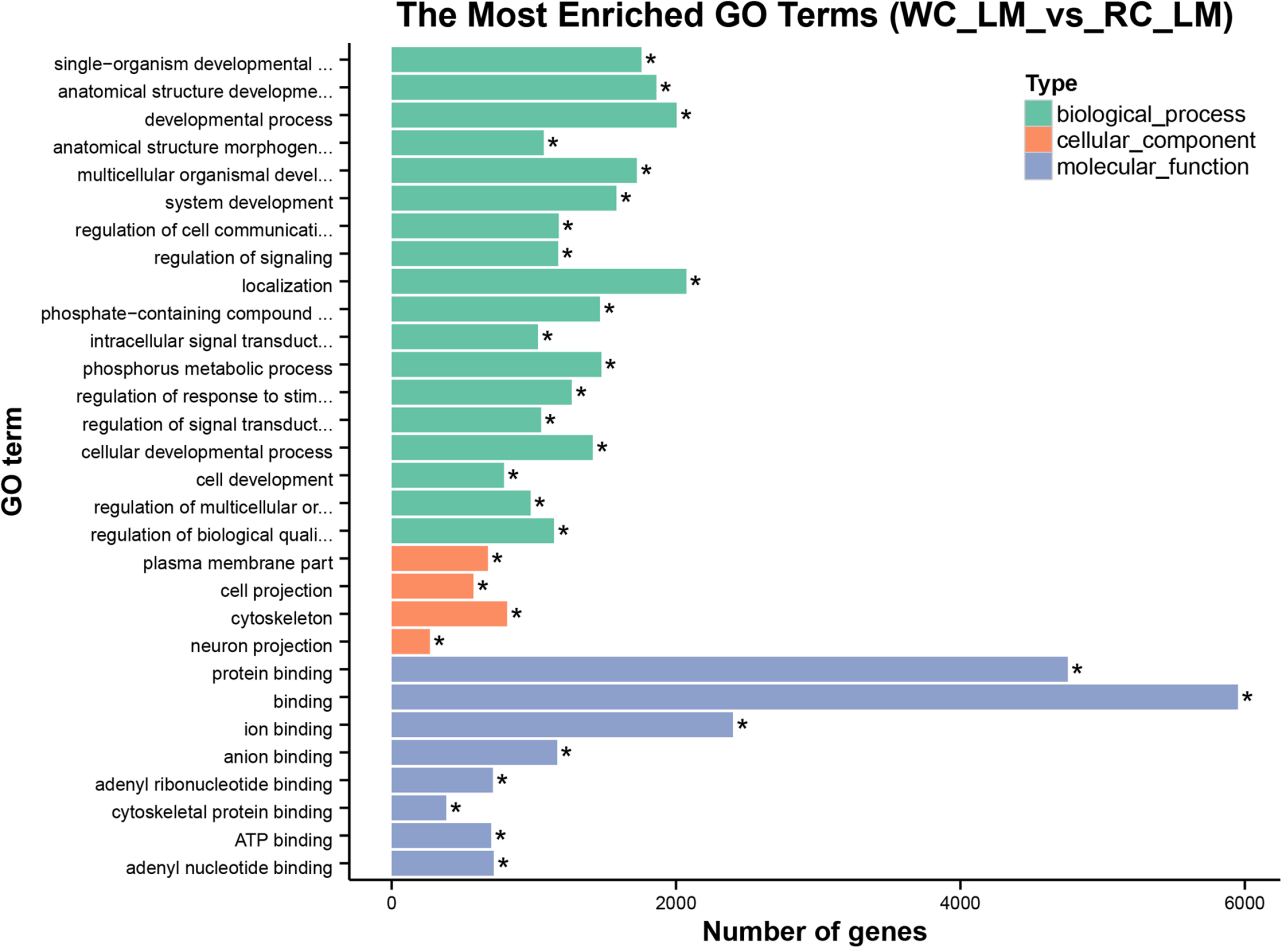
Histogram of enriched GO terms. The ordinate represents the enriched GO terms; the abscissa represents the number of genes; “*” represents the GO term significantly enriched (corrected *p*< 0.05) (only top 30 GO terms are listed by ascending order of corrected *p* values).

The identified DMRs were functionally classified by GO analysis. Functional enrichments on 23,150 DMRs (corrected *p*< 0.05) by gene ontology analysis found that 8,596 genes related to DMRs were enriched in 9,922 GO terms, among which 1,046 GO terms were significantly enriched (corrected *p*< 0.05). The most significant functional terms were binding (GO: 0005488) of hyper-methylated genes and protein binding (GO: 0005515) of hypo-methylated genes. In terms of biological process, the most significant functional terms were localization (GO: 0051179) of hyper-methylated genes and anatomical structure morphogenesis (GO: 0009653) of hypo-methylated genes. In cellular component, the most significant functional terms were plasma membrane parts (GO: 0044459) of hyper-methylated genes and cell projection (GO: 0042995) of hypo-methylated genes. The top 30 GO terms were listed in Fig 4 by ascending order of corrected *p* value.

**Fig 5:**
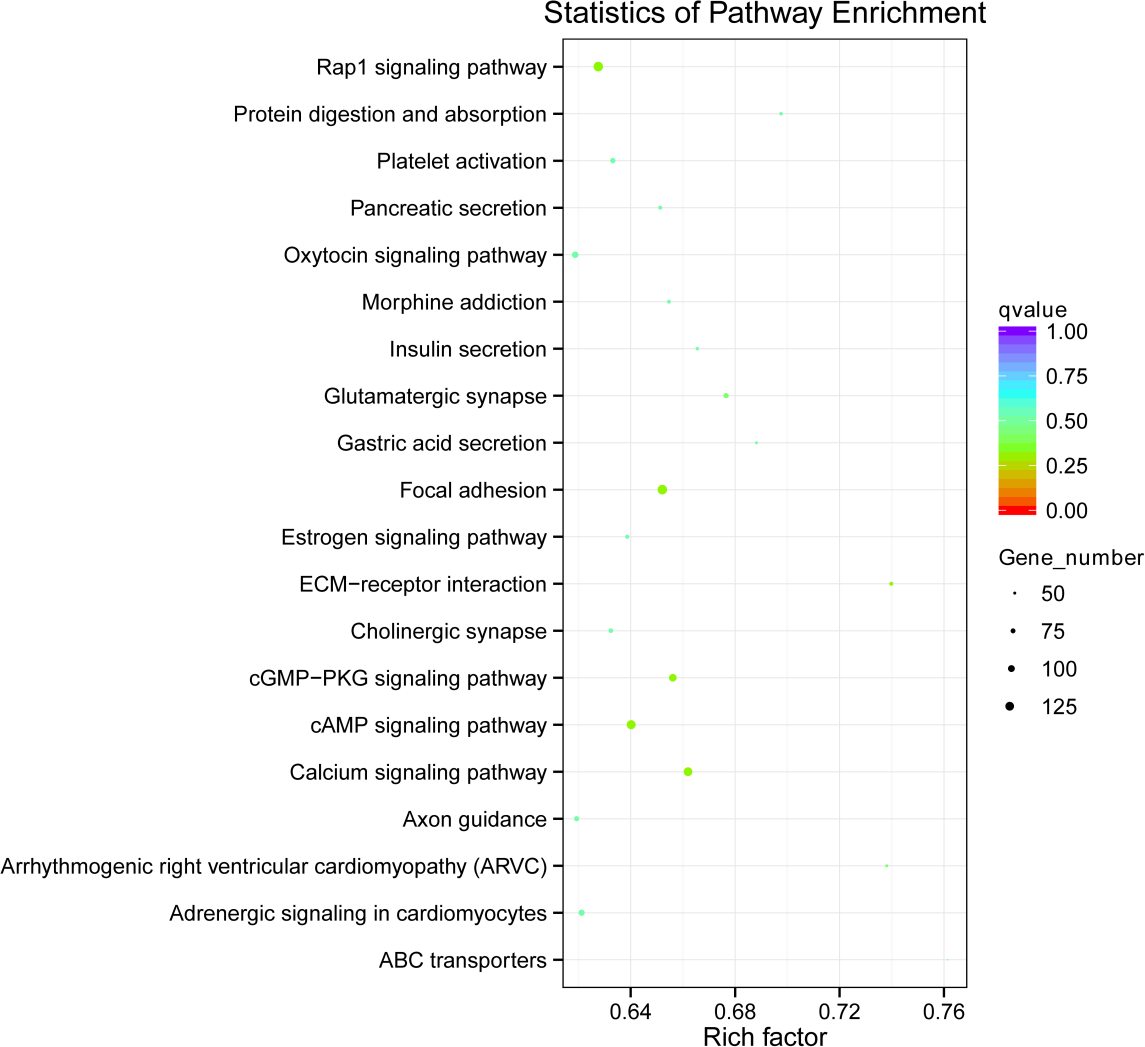
Scatterplot of enriched KEGG pathways. The ordinate represents the enriched pathways, and the abscissa represents the rich factor of corresponding pathways; the size of the spots represents the number of genes related to DMRs enriched in each pathway, while the color of the spot represents the corrected *p* value of each pathway. The rich factors indicate the ratio of the number of DMGs mapped to a certain pathway to the total number of genes mapped to this pathway. Greater rich factor means greater enrichment. DMRs: differentially methylated regions; DMGs: differentially methylated genes.

KEGG enrichment analysis of genes related to DMRs was also conducted for both hyper-methylated and hypo-methylated genes. Overall, 276 pathways were identified, such as calcium signaling pathway (bta04020), focal adhesion (bta04510), cAMP signaling pathway (bta04024), and cGMP-PKG signaling pathway (bta04022), and the top 20 in ascending order of corrected *p* value were listed in Fig 5. Results showed that calcium signaling pathway (bta04020, corrected *p* = 0.002), glutamatergic synapse (bta04724, corrected *p* = 0.047) and cAMP signaling pathway (bta04024, corrected *p* = 0.047) were significantly enriched in hyper-methylated genes (corrected *p*< 0.05), with calcium signaling pathway showed the highest enrichment (S4 Fig). However, no pathway was significantly enriched in hypo-methylated genes (corrected *p*> 0.05).

### Association analysis between DMRs and DEGs

**Fig 6:**
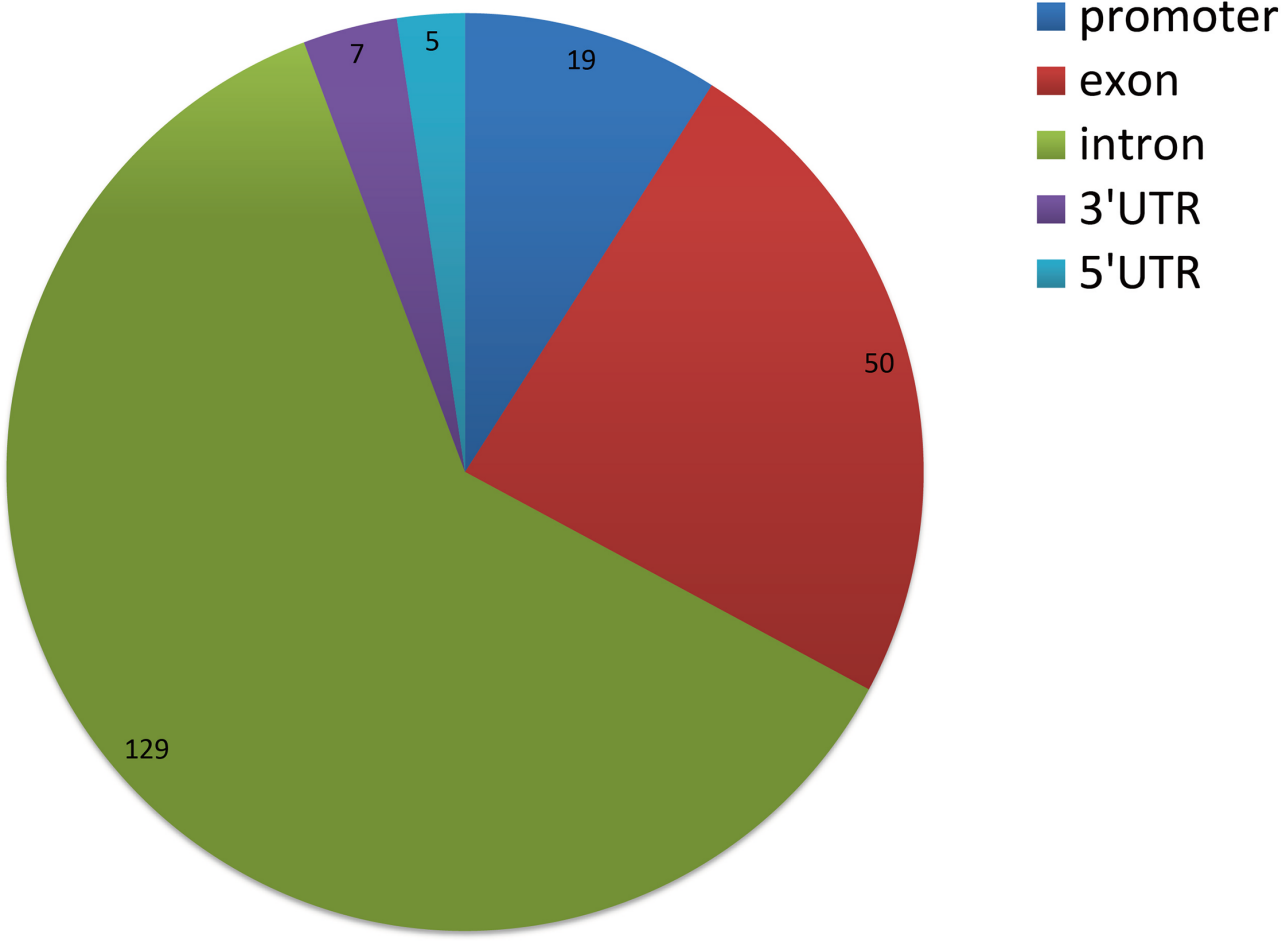
Distribution among different genomic elements of DEGs with expression level negatively correlated with DMRs methylation level. DMRs: differentially methylated regions; DEGs: differentially expressed genes.

**Fig 7:**
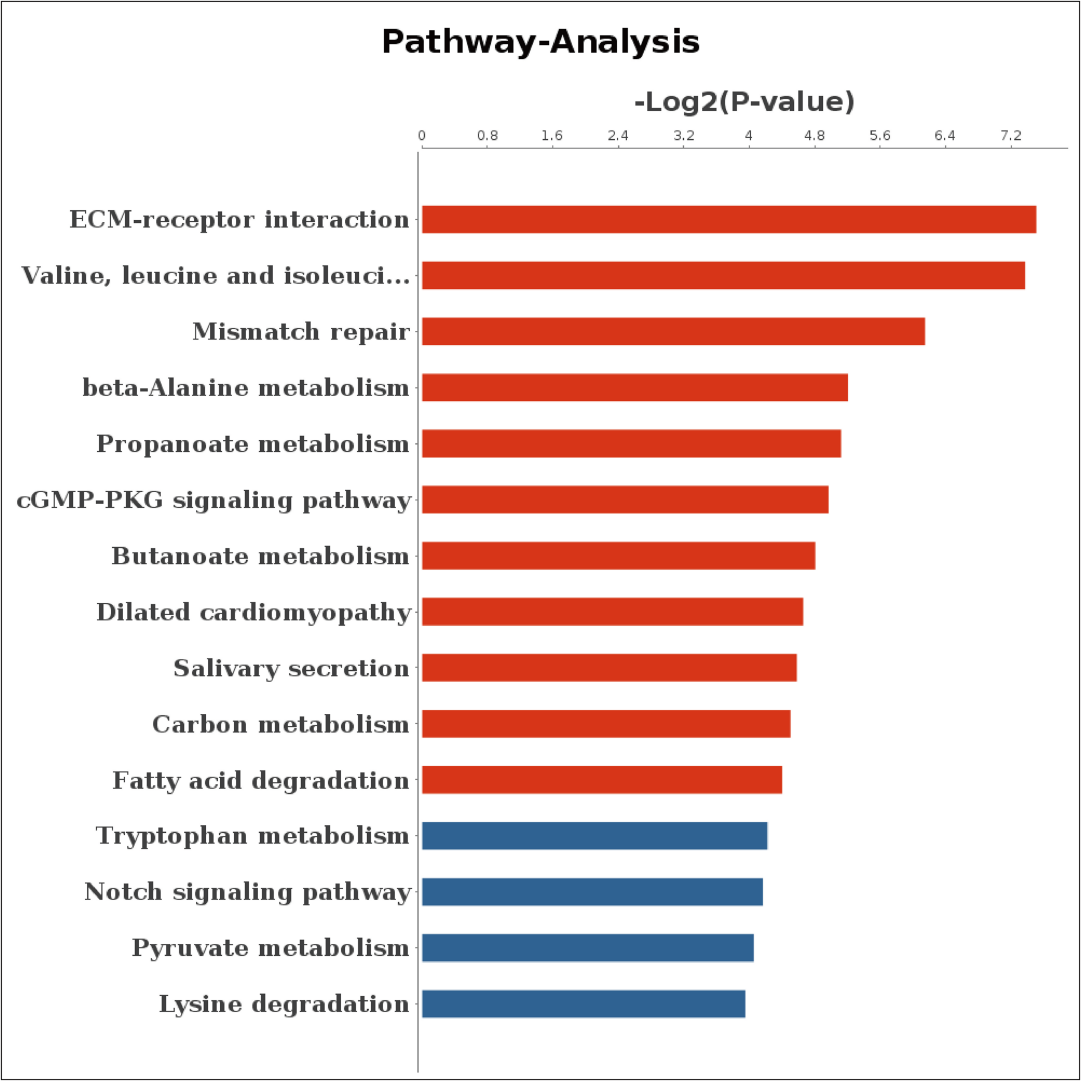
Pathway enrichment of DEGs with expression level negatively correlated with DMRs methylation level. Abscissa represents the value of log2 (*p* value) and ordinate represents the pathway name; Red bars indicate corrected *p*< 0.05. DMRs: differentially methylated regions; DEGs: differentially expressed genes.

We next analyzed the correlation between the methylation levels of DMRs and the expression levels of DEGs. The results indicated that 147 DMR related genes had negative correlation with the expression levels of DEGs (Table 5), of which 19 genes had DMRs in promoter regions, 50 genes had DMRs in exons, 129 genes had DMRs in introns and only 7 genes and 5 genes had DMRs in 3’UTRs and 5’UTRs, respectively (Fig 6). For genes with negative correlation, 56 genes were enriched in 969 GO terms, of which 259 GO terms were significant (corrected *p*< 0.05) including positive regulation of fat cell differentiation (GO: 0045600, corrected *p* = 0.005), glycerol metabolic process (GO: 0006071, corrected *p* = 0.010), negative regulation of low-density lipoprotein particle receptor catabolic process (GO: 0032804, corrected *p* = 0.016), triglyceride metabolic process (GO: 0006641, corrected *p* = 0.046), negative regulation of lipoprotein lipase activity (GO: 0051005, corrected *p* = 0.031) and glycerophospholipid metabolic process (GO: 0006650, corrected *p* = 0.039). In addition, the 56 genes were enriched in 118 pathways, of which 11 pathways were significant (corrected *p*< 0.05) such as ECM-receptor interaction (bta04512, corrected *p* = 0.005), propanoate metabolism (bta00640, corrected *p* = 0.029), cGMP-PKG signaling pathway (bta04022, corrected *p* = 0.032), carbon metabolism (bta01200, corrected *p* = 0.044), fatty acid degradation (bat00071, corrected *p* = 0.047) (Fig 7). Interestingly, 13 genes with negative correlation were enriched in metabolic pathway term (bta01100). Among negatively correlated genes, 6 genes with DMRs in promoter region were enriched in 10 pathways in which 4 pathways were significantly enriched (corrected *p*< 0.05) (S5 Fig).

**Table 5:** DEGs with gene expression level negatively correlated with DMRs methylation level.

Gene ID	Gene context	Gene Name
ENSBTAG00000005744	exon, intron	COQ2
ENSBTAG00000017661	exon, intron	RFX2
ENSBTAG00000019448	exon, intron, utr5	CLDN7
ENSBTAG00000007319	exon, intron	JAG2
ENSBTAG00000021024	exon, intron	MACF1
ENSBTAG00000010851	exon	SEPHS2
ENSBTAG00000018966	exon, intron	PLCE1
ENSBTAG00000019162	exon, intron	RNMTL1
ENSBTAG00000015794	exon	NES
ENSBTAG00000021457	exon, intron	EFEMP2
ENSBTAG00000019517	exon, intron	ELN
ENSBTAG00000021919	exon, intron	NAV1
ENSBTAG00000038439	exon, utr5, promoter	H1F0
ENSBTAG00000021381	exon, intron, utr3	DAAM2
ENSBTAG00000019569	exon, intron, utr5	CD151
ENSBTAG00000017574	exon, intron	LMNA
ENSBTAG00000000072	exon, intron	TFB2M
ENSBTAG00000030258	exon, intron	CDC42EP1
ENSBTAG00000021672	exon, utr3	RGS1
ENSBTAG00000005083	exon, intron	TULP4
ENSBTAG00000005762	exon, intron, utr5, promoter	LYNX1
ENSBTAG00000016269	exon, intron	ME2
ENSBTAG00000010682	promoter, exon, intron	DDR1
ENSBTAG00000003786	exon, intron	GCGR
ENSBTAG00000012505	exon, intron	ARHGEF17
ENSBTAG00000019375	exon, intron	SCARF2
ENSBTAG00000014885	exon, intron	MYOM3
ENSBTAG00000010402	exon, intron	MYH9
ENSBTAG00000018629	exon, intron	CWF19L2
ENSBTAG00000019967	exon, intron	RASA3
ENSBTAG00000006187	exon, intron	MFAP4
ENSBTAG00000004283	exon, intron	PPFIBP1
ENSBTAG00000038844	exon	ANKRD35
ENSBTAG00000003619	exon, intron	SEC24D
ENSBTAG00000013334	exon, intron	CSF3R
ENSBTAG00000013869	exon, intron, utr3	SH3BP4
ENSBTAG00000012849	exon, intron	COL4A1
ENSBTAG00000013004	exon, utr3, intron	ITIH5
ENSBTAG00000030340	exon, utr3, intron	-
ENSBTAG00000008518	exon, intron	MED25
ENSBTAG00000033835	exon, intron, utr3	MPZ
ENSBTAG00000037383	exon, intron	AKAP13
ENSBTAG00000000961	exon, utr3	NEURL2
ENSBTAG00000000054	promoter, exon, intron	SNAPC4
ENSBTAG00000006213	exon, utr5, promoter	IFT27
ENSBTAG00000017528	exon, intron	SNAI3
ENSBTAG00000002024	exon, intron	ASB18
ENSBTAG00000001396	exon, intron	ADAMTS7
ENSBTAG00000009733	exon, intron	FBP1
ENSBTAG00000019002	exon, intron	SLC2A12

DMRs: differentially methylated regions; DEGs: differentially expressed genes; gene context: functional region names; promoter: the 2kb region upstream of the TSS point.

## Discussion

DMR is an important epigenetic marker which may be involved in regulation of gene expression by changing chromatin structure or transcription efficiency under different conditions [24]. Although DNA methylation analysis has been performed on cattle [12, 25, 26], this study is the first to systematically compare the genome-wide methylation profiles in longissimus dorsi muscle between Japanese black cattle and Chinese Red Steppes, between which significant difference exist in overall meat quality traits.

### DNA methylation profiles in longissimus dorsi muscle

The genome-wide methylation patterns in functional genomic regions were very similar between the two breeds. However, there were differences among three mC contexts which might be related to the sequence differences in different genetic elements. The majority of DMRs were small fragments (50–1000 nt > 90% of DMRs), which suggested that methylation changes within a small regions could play an important role on the regulation of gene expression. There were more DMRs with higher methylation level and less DMRs with lower methylation in Wagyu in comparison to Chinese Red Steppes, and DMRs were mainly concentrated in the intron regions (> 70%) with only a small proportion distributed in the 3’ UTRs and 5’ UTRs. In addition, the distribution of certain DMRs in either hyper-methylated or hypo-methylated status with the same gene indicated that different methylation status in different regions might have different regulatory functions on gene expression.

### Correlation analysis between DMRs and signal pathways

Analyses of pathways and genes related to DMRs revealed that the hyper-methylated genes were significantly concentrated in three pathways: calcium signaling pathway (bta04020, corrected *p* = 0.002), glutamatergic synapse (bta04724, corrected *p* = 0.047) and cAMP signaling pathway (bta04024, corrected *p* = 0.047) (S6 Fig). The KEGG enrichment analysis indicated that calcium signaling pathway was the most significantly correlated pathway, which is the key pathway exerting allosteric regulation on many proteins by the calcium ions signaling effects, such as activation of ion channels or as a secondary messenger.

It is reported that *Caveolin 1* (*CAV1*) gene has a higher expression level in adipocytes [27], and *CAV1* gene knockout leads to imbalance in lipid metabolism [28]. Phospholipase C (PLC) cleaves phospholipids into free fatty acids (FFA), lysophospholipids and diacylglycerol, and the relative activities of PLC are highly correlated with the decrease of phospholipids and the increase of free fatty acids [29]. These studies indicated that *CAV1* and *PLC* genes are involved in lipid and fatty acid metabolism. A mutation in the *ryanodine receptor* (*RyR*) gene is found to be correlated with malignant hyperthermia in lean, heavily muscled swine breeds [30]. In this study, *CAV1*, *PLC* and *RYR* genes were identified as differentially methylated genes between the two breeds, which suggested that certain differentially methylated genes in calcium signaling pathway could affect the metabolism of the skeletal muscles. Peroxisome proliferator-activated receptor gamma (*PPARγ*), a key molecule found in cAMP signal pathway involving the G protein coupled receptors [31], is involved in adipocyte differentiation and function. The present study identified three types of PPARs: alpha, gamma, and delta [32]. PPARγ is expressed in liver, kidney, heart, muscle, adipose tissue, and others [33]. As a dietary lipid sensor, the activation of PPARγ results in lipid metabolism in muscle [34]. Interestingly, all the three pathways could be regulated by calcium ions concentration, which indicated that this might be the core pathway in the regulation of muscle metabolism [35].

### Negative correlation between the methylation levels of DMRs and differential expressions of DEGs

There is a complex relationship between gene methylation and gene expression level. Current research data suggest that methylation in the promoter region blocks transcription initiation, but the function on gene expression of DNA methylation within gene body has not been clearly understood [24].

As seen in Table 5, the DMRs in the promoter and gene body regions were negatively correlated with gene expression level. *Enoyl-CoA*, *hydratase/3-hydroxyacyl CoA dehydrogenase* (*EHHADH*) and *4-N-trimethylaminobutyraldehyde dehydrogenase* (*ALDH9A1*) genes were enriched in the pathway of fatty acid degradation (*p* = 0.047, *p*< 0.05), during which fatty acids are broken down into acetyl-CoA to enter into the citric acid cycle for energy production in animals [36]. It seemed that DMRs related to genes in the fatty acid degradation pathway might affect fatty acid composition in meat through regulation of gene expression levels. EHHADH gene was identified by GWAS as one of the 20 promising novel genes associated with milk fatty acid traits in Chinese Holstein [37]. Furthermore, *EHHADH* and *angiopoietin-like protein 4* (*ANGPTL4*) genes both belong to the PPAR signaling pathway (bta03320), which is a key biological pathway in determining meat quality traits in mammals by involving in lipid metabolism. ANGPTL4 is mainly expressed in liver and adipose tissues [38] and plays important roles in the regulation of lipid metabolism. Previous studies have suggested that a SNP in ANGPTL4 is associated with intramuscular fat [39] and the expression level of ANGPTL4 affects meat quality traits of pigs [40]. Recent studies have shown that acyl-CoA synthetase family member 3 (ACSF3), in which DMRs in promoter region were found in the present study to be negatively correlated with the gene expression level, plays a critical role in mammal fatty acid synthesis in mitochondrion [41]. Thus EHHADH, ALDH9A1, ANGPTL4 and ACSF3 play important roles in fatty acid and lipid metabolism in muscle and regulation of these genes through DNA methylation might be part of the mechanisms in determining the difference in meat quality traits between the two breeds.

The classical theory on animal breeding believes that the appearance of a quantitative trait is the result of multiple genetic loci, in particular, a single polymorphic locus with multiple, differentially expressed alleles can give rise to the quantitative trait variation within a natural population [42]. Many of the differentially methylated genes identified in the present study between Wagyu and Chinese Red Steppes are also found to be differentially methylated in genes related to lipid metabolism and fatty acid metabolism, such as *fatty acid synthase* (*FASN*) which is shown to be related to fatty acid composition in a genome-wide association study of Japanese black cattle [43], and *carnitine palmitoyltransferase 1A* (*CPT1A*), *fatty acid desaturase 1* (*FADS1*) and *acyl-CoA synthetase long-chain family member 1* (*ACSL1*) genes which are well-known genes in the pathways of fatty acid metabolism and fatty acid degradation. In addition, other genes known to affect meat quality traits of cattle are also related to the lipid metabolism and fatty acid metabolism (*insulin like growth factor 2* (*IGF2*), *leptin* (*OB*), *lipase hormone sensitive* (*HSL*), *diacylglycerol O-acyltransferase 1* (*DGAT1*) and *fatty acid binding protein 3* (*FABP3*)) [44–49]. We believed that the methylation of these genes might partially contribute to the significant variance in meat quality traits between Japanese black cattle and Chinese Red Steppes. However, the regulatory epigenetic mechanisms of these genes and genetic regions on bovine meat quality traits require further study.

## Conclusion

In the present study, whole genome, high resolution DNA methylation profiles of longissimus dorsi muscles were established for Japanese black and Chinese Red Steppes cattle. Combined with transcriptomic analysis, our study investigated the genome-wide regulation of gene expression by DNA methylation at transcription level, and identified a number of novel and important genes associated with DMRs and pathways that might affect muscle development and meat quality traits in cattle, which needed to be further experimentally validated in the future. Our results provided valuable data to further our understanding of the genetic and epigenetic mechanisms that control economic traits in cattle, which could be used in marker-assisted selection programs to improve beef production.

## Supporting information

S1 Fig Distribution of genomic sequencing depth. (A) The distribution of sequence coverage at the genome of all samples, abscissa represents the depth of coverage and ordinate represents its frequency. (B) The accumulated distribution of sequence coverage at genome of all samples, abscissa represents the depth of coverage and ordinate represents the ratio of coverage not less than the depth of the total number of base sites; different lines with different colors represent different samples.
(TIF)

S2 Fig Clusters of different functional regions by methylation levels. (TIF)

S3 Fig Distribution of DMR lengths. (TIF)

S4 Fig Pathways enriched for genes related to DMRs with higher methylation levels in Japanese black cattle (Wagyu) than that in Chinese Red Steppes. The ordinate represents the enriched pathways, and the abscissa represents the rich factor of corresponding pathways; the size of the spots represented the number of genes related to DMRs enriched in each pathway, while the color of the spot represents the corrected *p* value of each pathway. The rich factors indicate the ratio of the number of DMGs mapped to a certain pathway to the total number of genes mapped to this pathway. Greater rich factor means greater enrichment. DMRs: differentially methylated regions; DMGs: differentially methylated genes.
(TIF)

S5 Fig Pathway enrichment of genes with negative correlation between methylation levels of DMRs in promoter and expression levels of mRNA. Abscissa represents the value of log2 (*p* value) and ordinate represents the pathway name; red bars indicate corrected *p*< 0.05.
(TIF)

S6 Fig KEGG pathways with genes related to significantly enriched DMRs with higher methylation in Wagyu. Genes with red marker are related to DMRs with higher methylation levels in Japanese black cattle (Wagyu) than that in Chinese Red Steppes. DMRs: differentially methylated regions.
(TIF)

S1 Table Comparison of DNA methylation patterns between the two groups. (DOCX)
