# Supplementary figures and images for: Comparative genome-wide methylation analysis of longissimus dorsi muscles between Japanese black (Wagyu) and Chinese Red Steppes cattle
Source: PLoS One. 2017 Aug 3;12(8):e0182492. doi: 10.1371/journal.pone.0182492 (PMC5542662; doi:10.1371/journal.pone.0182492)

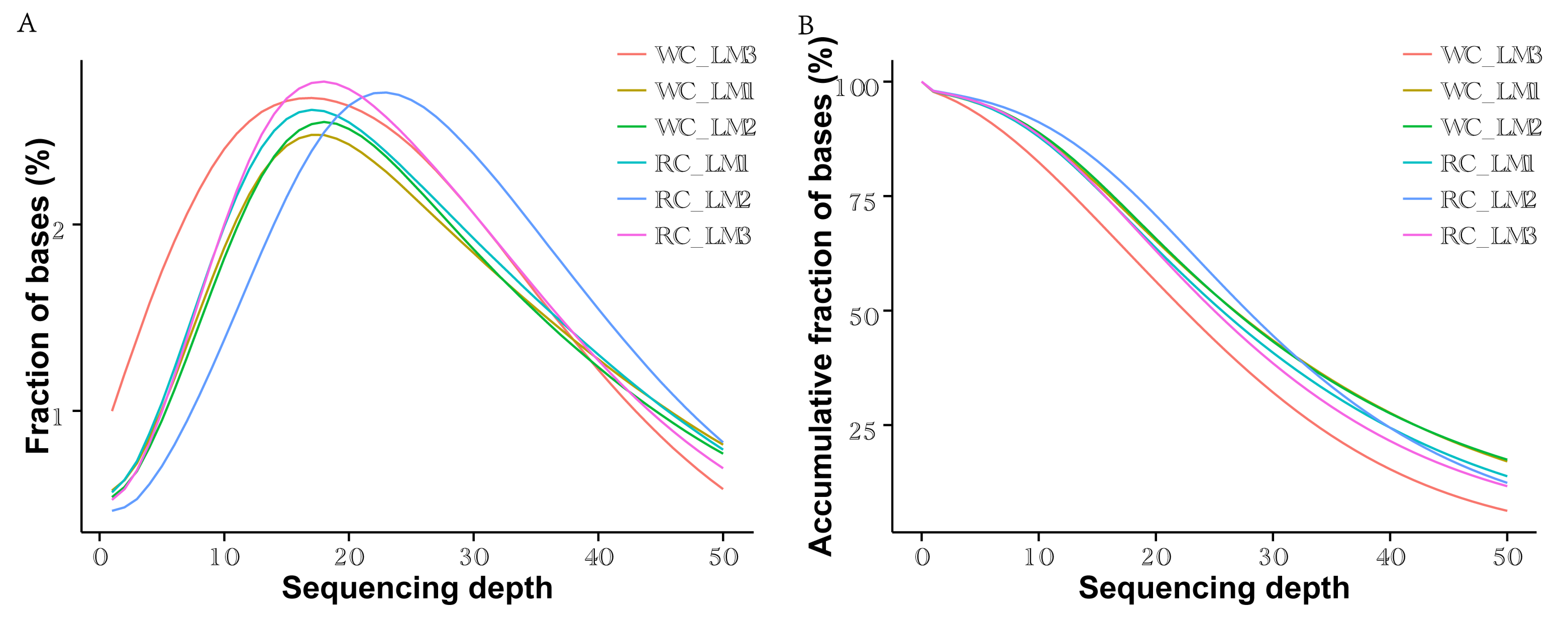

Supplement: S1 Fig — (A) The distribution of sequence coverage at the genome of all samples, abscissa represents the depth of coverage and ordinate represents its frequency. (B) The accumulated distribution of sequence coverage at genome of all samples, abscissa represents the depth of coverage and ordinate represents the ratio of coverage not less than the depth of the total number of base sites; different lines with different colors represent different samples. (TIF) [file pone.0182492.s001.tif]

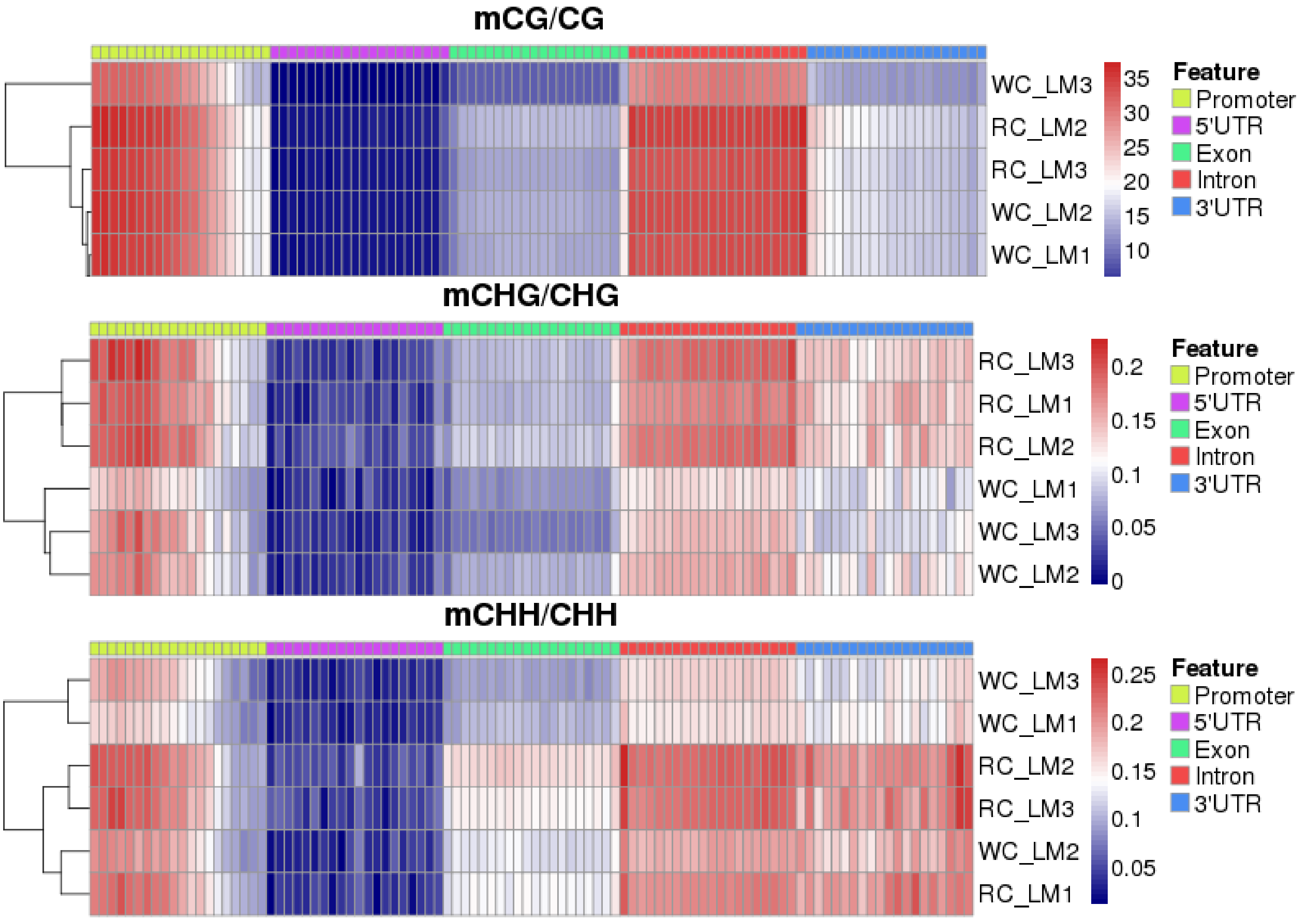

Supplement: S2 Fig — (TIF) [file pone.0182492.s002.tif]

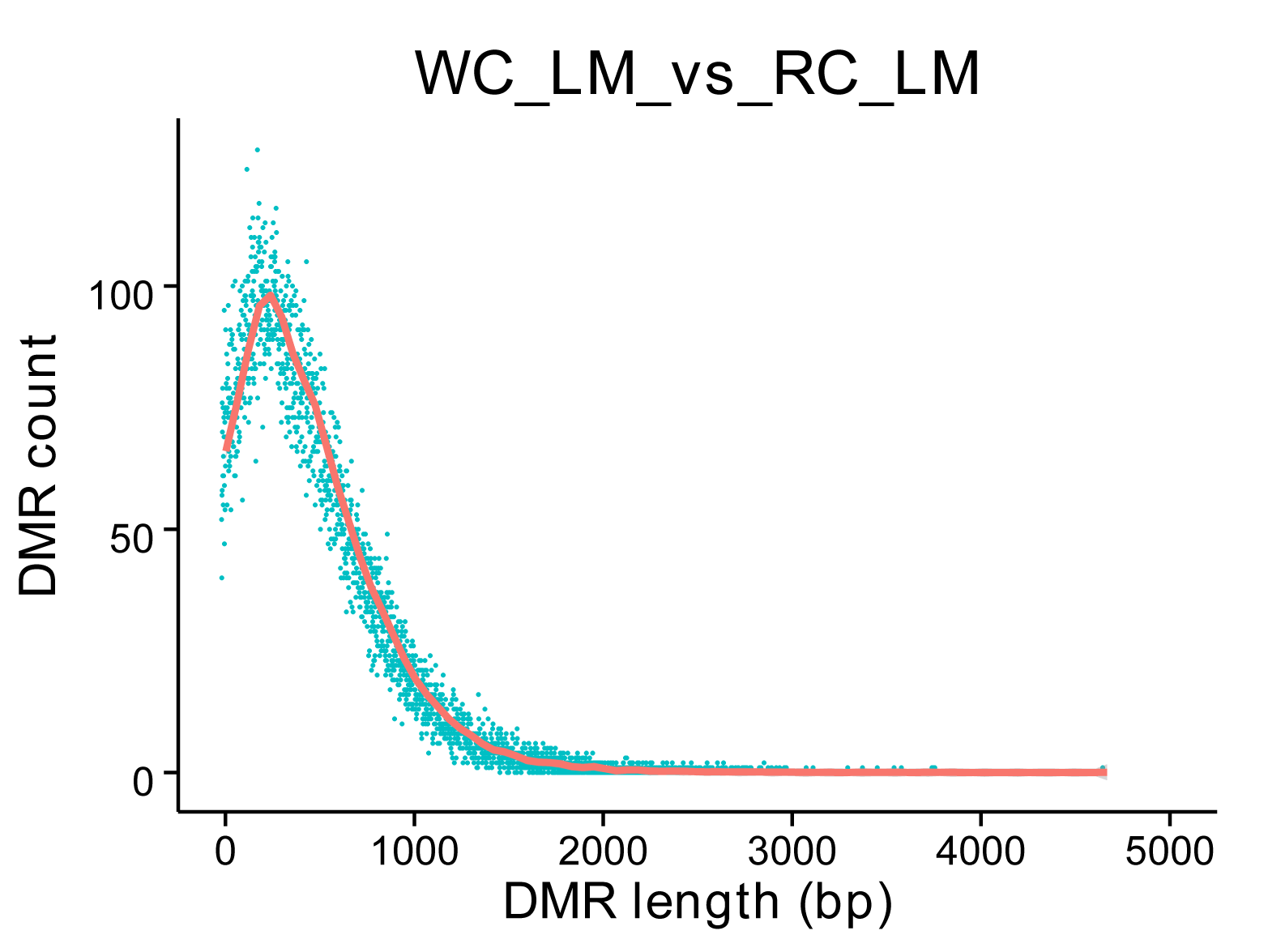

Supplement: S3 Fig — (TIF) [file pone.0182492.s003.tif]

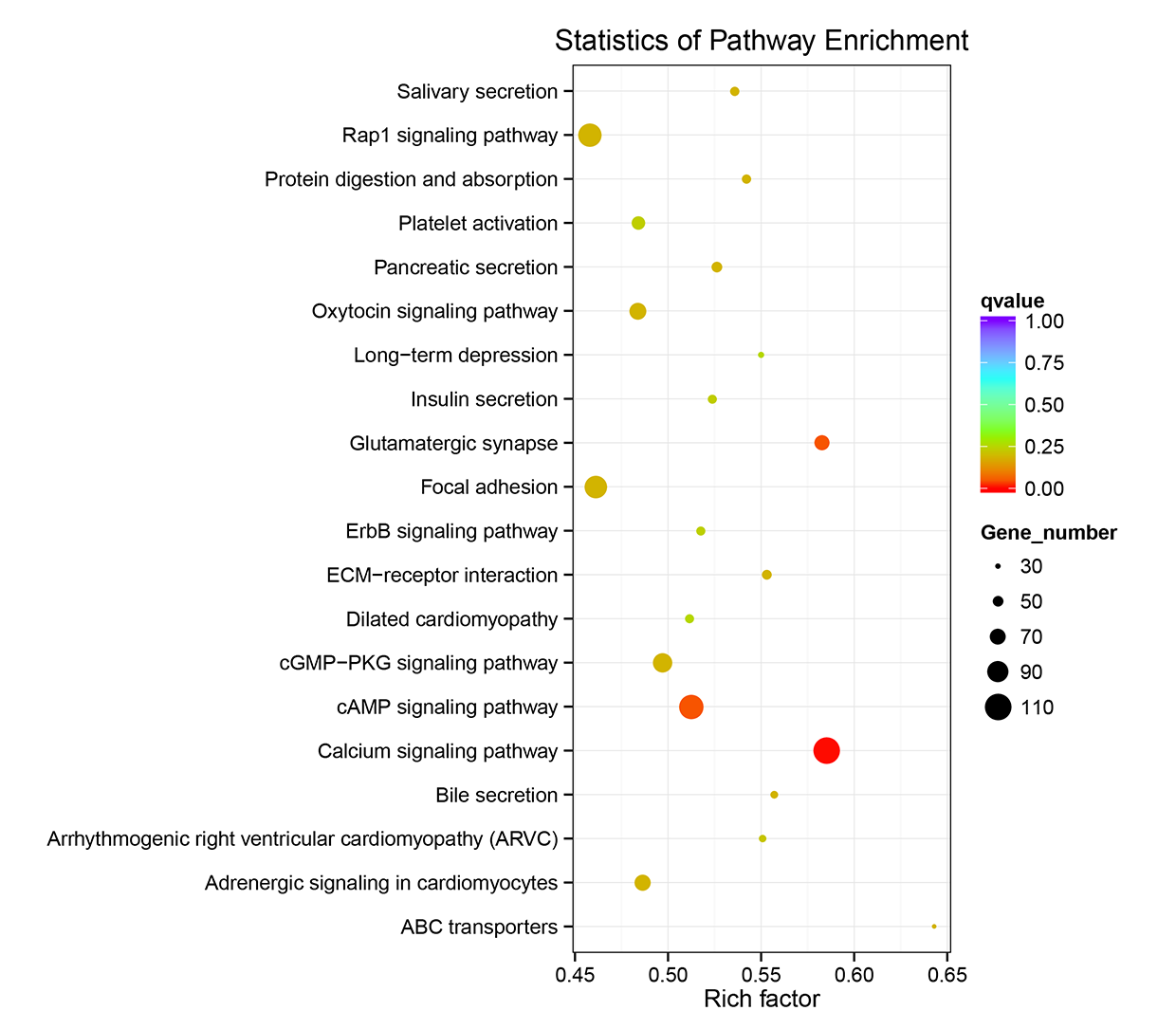

Supplement: S4 Fig — The ordinate represents the enriched pathways, and the abscissa represents the rich factor of corresponding pathways; the size of the spots represented the number of genes related to DMRs enriched in each pathway, while the color of the spot represents the corrected p value of each pathway. The rich factors indicate the ratio of the number of DMGs mapped to a certain pathway to the total number of genes mapped to this pathway. Greater rich factor means greater enrichment. DMRs: differentially methylated regions; DMGs: differentially methylated genes. (TIF) [file pone.0182492.s004.tif]

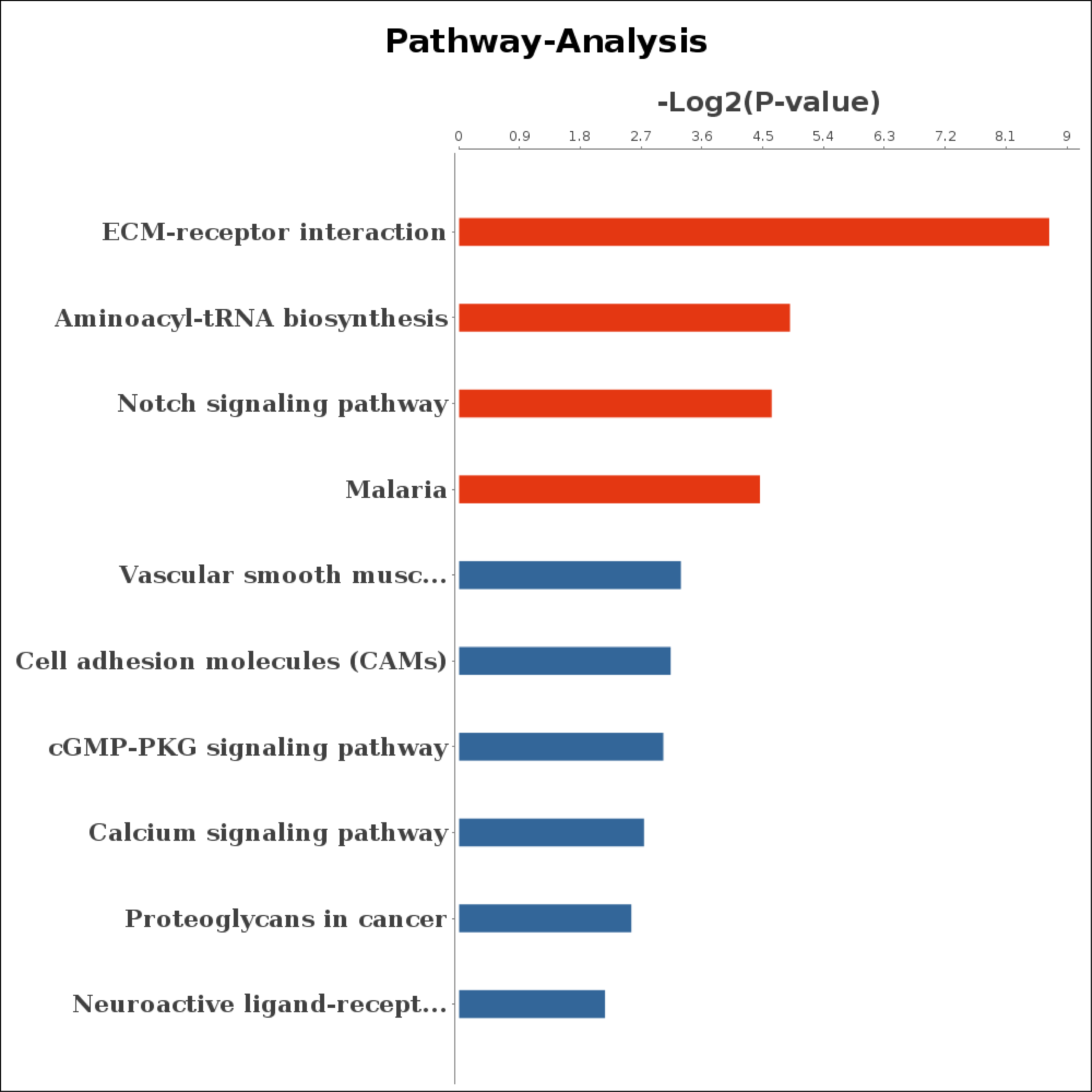

Supplement: S5 Fig — Abscissa represents the value of log2 (p value) and ordinate represents the pathway name; red bars indicate corrected p< 0.05. (TIF) [file pone.0182492.s005.tif]

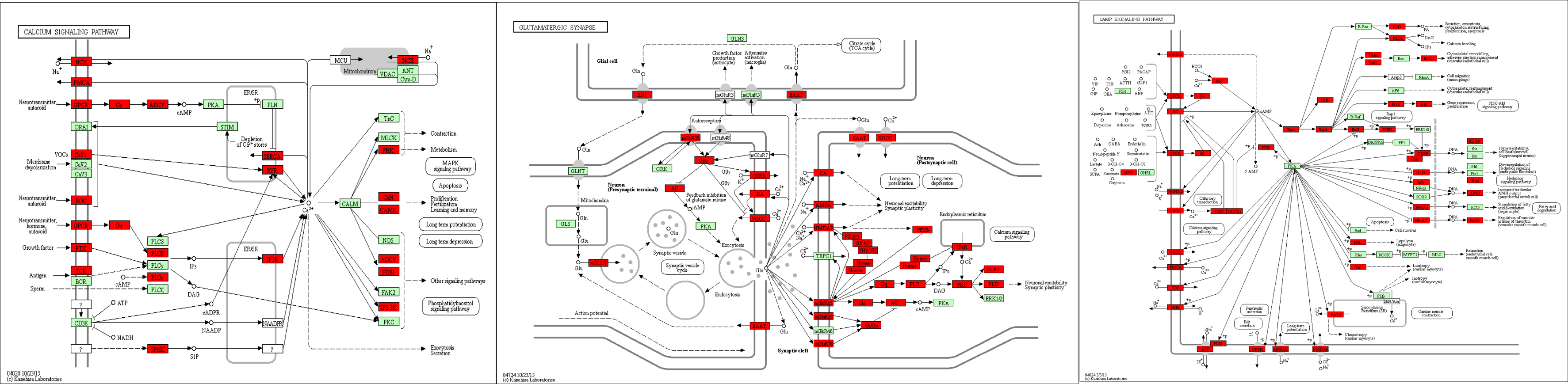

Supplement: S6 Fig — Genes with red marker are related to DMRs with higher methylation levels in Japanese black cattle (Wagyu) than that in Chinese Red Steppes. DMRs: differentially methylated regions. (TIF) [file pone.0182492.s006.tif]
