# Supplementary material for: Comparative genome-wide methylation analysis of longissimus dorsi muscles between Japanese black (Wagyu) and Chinese Red Steppes cattle
Source: PLoS One. 2017 Aug 3;12(8):e0182492. doi: 10.1371/journal.pone.0182492 (PMC5542662; doi:10.1371/journal.pone.0182492)
Supplement: S1 Table — (DOCX) [file pone.0182492.s007.docx]

**Supplementary Tables**

**S1 Table. Comparison of DNA methylation patterns between the two groups.**

| **Samples** | **mC** | **mCG** | **mCHG** | **mCHH** |
| --- | --- | --- | --- | --- |
| WC_LM3 | 27256896 (100%) | 26261984 (96.35%) | 165795 (0.61%) | 829117 (3.04%) |
| WC_LM1 | 31726921 (100%) | 30870250 (97.30%) | 175641 (0.55%) | 681030 (2.15%) |
| WC_LM2 | 32348366 (100%) | 31337956 (96.88%) | 196444 (0.61%) | 813966 (2.52%) |
| RC_LM1 | 31782665 (100%) | 30640302 (96.41%) | 208016 (0.65%) | 934347 (2.94%) |
| RC_LM2 | 36430010 (100%) | 35153315 (96.50%) | 242673 (0.67%) | 1034022 (2.84%) |
| RC_LM3 | 32119064 (100%) | 30909958 (96.24%) | 228866 (0.71%) | 980240 (3.05%) |

Samples: sample names; mC: number and percentage of methylated cytosine in whole genome (considered as 100%); mCG: numbers of methylated cytosine in CG context and percentage of methylated cytosine in whole genome; mCHG: numbers of methylated cytosine in CHG context and percentage of methylated cytosine in whole genome; mCHH：numbers of methylated cytosine in CHH context and percentage of methylated cytosine in whole genome (H=A, T or C).
